# Combination of single-cell and bulk RNA-seq reveals changes in the immune landscape in osteomyelitis

**DOI:** 10.3389/fimmu.2026.1746323

**Published:** 2026-02-27

**Authors:** Zhenhua Zhu, Xiaopeng Jing, Jun Chen, Siteng Li, Jie Tan, Ji Zeng, Zheng Jin

**Affiliations:** 1 Department of Joint Surgery, Wuhan Fourth Hospital, Wuhan, China; 2 Department of Clinical Laboratory, Wuhan Fourth Hospital, Wuhan, China; 3 Orthopedic Department, Wuhan Fourth Hospital, Wuhan, China; 4 School of Medicine, Nankai University, Tianjin, China; 5 Department of Orthopaedic Trauma, Center for Orthopaedic Surgery, The Third Affiliated Hospital, Southern Medical University, Guangzhou, China; 6 Orthopedic Laboratory, Orthopedic Department and Hubei Sports Medicine Center, Wuhan Fourth Hospital, Wuhan, China

**Keywords:** gene expression, immune cell infiltration, macrophages, osteomyelitis, single-cell sequencing, therapeutic targets, transcriptomic analysis

## Abstract

**Objective:**

This study presented a comprehensive characterization of the osteomyelitis immune microenvironment, identified driver genes and pathogenic cell populations underlying disease progression, and uncovered potential therapeutic targets through single-cell and bulk transcriptomic analysis.

**Methods:**

We analyzed time-series transcriptomic sequencing data from mouse osteomyelitis samples in the dataset GSE168896. Fuzzy c-means clustering was applied to reveal gene sets linked to disease progression. Immune cell infiltration analysis was conducted through the online tool ImmuCellAI-mouse. Furthermore, by leveraging single-cell sequencing data, we characterized immune cell subpopulations and pinpointed the key cell subtypes that were present in the osteomyelitis mice.

**Results:**

We identified six gene clusters exhibiting distinct temporal expression patterns and functional roles in osteomyelitis, such as leukocyte and lymphocyte activation and ossification. Single-cell sequencing analysis further showed seven distinct cellular subpopulations. Among these, macrophages demonstrated a significant increase following osteomyelitis, and the infiltration of Mif^+^Cd63^+^, Arg1^+^Sdc4^+^, and Cxcl1^+^Ccl4^+^ macrophages significantly increased. Moreover, Ccl3–Ccr1 and Cxcl2–Cxcr2 ligand–receptors contributed mostly in immune cells.

**Conclusion:**

Our findings tracked the transcriptional dynamics and evolving immune landscape of osteomyelitis, highlighting macrophages as central regulators of disease progression. We identified that significant infiltration of Arg1^+^Sdc4^+^, Cxcl1^+^Ccl4^+^, and Mif^+^Cd63^+^ macrophages may affect osteomyelitis through the Ccl3–Ccr1 and Cxcl2–Cxcr2 signaling pathways. These findings offer a new perspective on immune regulation in osteomyelitis.

## 1 Introduction

Osteomyelitis is characterized by infection and inflammation of the bone and surrounding tissues, most commonly caused by bacterial or fungal pathogens (1, 2). The management of osteomyelitis continues to present a significant clinical challenge in orthopedics (3), with approximately 10%–30% of acute infections progressing to chronic osteomyelitis (4). Despite advancements in antibiotic therapies and surgical techniques, the long-term recurrence rate remains as high as 20% (5), primarily due to factors such as pathogen resistance, biofilm formation, and severe bone tissue damage (6).

Immunity shapes how osteomyelitis unfolds, either restraining or accelerating its course, acting as both a defender against infection and a potential contributor to bone tissue damage (7, 8). The body’s first shield is innate immunity, which spots pathogen-associated molecular patterns (PAMPs) via pattern recognition receptors (PRRs) (9). For example, components such as lipoteichoic acid and peptidoglycan from *Staphylococcus aureus* (*S. aureus*) interact with PRRs, initiating a signaling cascade that triggers an inflammatory call-to-arms, summoning immune cells to the infected zone (10). Early in an acute osteomyelitic episode, interleukin-8 (IL-8) acts as a critical chemotactic signal, recruiting neutrophils, monocytes, and macrophages to the site of infection. Neutrophils play a central role, actively phagocytosing pathogens and releasing potent effector molecules, including reactive oxygen species (ROS) and antimicrobial peptides (11). However, excessive neutrophil activity can cause collateral damage to bone and nearby tissues (12), worsening the pathology of osteomyelitis. Macrophages exist in different activation states, typically grouped into two subsets: a pro-inflammatory phenotype termed M1 and an anti-inflammatory phenotype termed M2. During osteomyelitis, the inflammatory response disrupts and delays the normal bone-healing process (13). The adaptive immune response, orchestrating the mobilization of T and B lymphocytes, generates a defense that is both highly antigen-specific and capable of establishing long-term immunological memory. Among T cells, the Th cells—especially the Th1 and Th17 subpopulations—act as key drivers in the clearance of invasive bacteria (14). Th1 cells release interferon-gamma (IFN-γ), a cytokine that turns macrophages into potent microbe killers (15), while Th17 cells produce IL-17, enhancing neutrophil recruitment (16). However, elevated levels of IL-17 can overactivate osteoclasts, which are responsible for bone resorption, leading to bone destruction (17). Immune-mediated inflammation is a primary driver of bone destruction in osteomyelitis. Pro-inflammatory cytokines promote osteoclast formation by upregulating receptor activator of nuclear factor κB ligand (RANKL) (18). RANKL latches onto receptor activator of nuclear factor-κB (RANK) on osteoclast precursors, driving their differentiation and activation (19), which in turn increases bone resorption and can result in significant bone loss. This dual role of the immune system (protective and pathological) presents opportunities for innovative treatment strategies. For example, cytokine-blocking therapies targeting pro-inflammatory cytokines may reduce tissue damage while preserving immune function (20). Clinical trials of anti-IL-17 therapies in other inflammatory conditions like rheumatoid arthritis (21) suggest that such approaches could also be effective in treating osteomyelitis. Additionally, therapies that reprogram macrophages—shifting them from a pro-inflammatory M1 state to an anti-inflammatory M2 state—could help clear infection and speed tissue healing (22).

At present, researches on the immune microenvironment in osteomyelitis still face numerous challenges, particularly in understanding the functions, diversity, and dynamic changes of immune cells (23). Traditional research methods, such as immunohistochemistry, flow cytometry, and ELISA, have contributed to advances in certain aspects. However, they exhibit the following limitations: 1) Limited analysis of immune cell phenotypes and functions: These methods often fall short in fully elucidating the activation states of immune cells or the diversity within their subpopulations. 2) Lack of a holistic immune network perspective: Traditional approaches struggle to uncover the intricate communication mechanisms between different immune cell types, making it difficult to capture the complexity of immune interactions. 3) Inadequate study of cellular heterogeneity: They are less effective in analyzing rare but functionally significant immune cell populations and often fail to track dynamic changes in tissue samples. 4) Insufficient temporal tracking: These methods lack the ability to depict time-series dynamic shifts in the local immune landscape during the progression of osteomyelitis. Emerging RNA sequencing technologies offer solutions to these limitations by providing high-resolution insights into genome-wide and single-cell expression profiles (24). RNA-seq enables the capture of immune gene expression dynamics within osteomyelitis lesions, allowing for the identification of intercellular communication and signaling networks. Furthermore, scRNA-seq provides a powerful tool for analyzing the heterogeneity, functional states, and differentiation trajectories of immune cell subsets (25).

In this study, we began by tracking transcriptomes across a longitudinal series related to osteomyelitis to identify gene clusters that were closely associated with its progression. Functional annotation of these gene clusters was performed to explore the key biological processes underlying osteomyelitis progression. Subsequently, single-cell sequencing data from osteomyelitis models were analyzed, revealing 10 distinct immune cell subsets. By comparing the immune cell subsets between osteomyelitis specimens and control specimens and examining the expression patterns of the identified hub genes, we identified macrophage subsets strongly implicated in osteomyelitis progression. Further investigation of these cell subsets included pseudo-time trajectory analysis, cell–cell interaction analysis, and transcription factor regulatory network analysis. These approaches allowed us to delineate the differentiation trajectory of this subset, its interactions with other immune cell populations, and its potential regulatory targets. Through this multilevel analysis, we provide valuable insights into the immune microenvironment and identify novel mechanisms in the progression of osteomyelitis. These results deepen our insight into disease mechanisms.

In summary, this study presents a comprehensive dynamic gene expression landscape and an in-depth immune cell profile of osteomyelitis, highlighting how macrophages orchestrate the process. By integrating time-series transcriptome data, single-cell sequencing analysis, and functional regulatory networks, our results add new depth to how osteomyelitis unfolds at the molecular and cellular levels. Moreover, this research identifies potential therapeutic targets, offering valuable insights for therapies that could transform how osteomyelitis is treated and managed.

## 2 Methods

### 2.1 Bulk RNA-seq data processing

Raw sequencing reads from the GEO dataset GSE168896 were preprocessed using FastQC (v0.12.0) to assess quality. Low-quality bases were removed by TrimGalore (v0.4.5.1) with default settings. Subsequently, the high-quality reads were mapped to the mouse reference genome (GRCm39) and quantified using kallisto (26). DESeq2 was employed for identifying differentially expressed genes (DEGs). Genes with an adjusted *p*-value (*p*adj) <0.05 were defined as DEGs. Fuzzy c-means clustering of dynamic genes was conducted using the R package “Mfuzz” (v2.66.0). The optimal number of clusters (*k* = 6) was determined via the minimum centroid distance criterion, with a fuzzification parameter (*m*) set to 1.25. Gene ontology (GO) enrichment for modules was performed using clusterProfiler (v4.14.4) with a threshold of *q*-value <0.05. The online tool immune cell abundance identifier (https://guolab.wchscu.cn/ImmuCellAI) was used for immune cell infiltration analysis with default parameters.

### 2.2 Constructing the mouse model of osteomyelitis

All animal procedures received prior approval from the Wuhan Fourth Hospital Institutional Animal Care and Use Committee. Mice were subjected to anesthesia and disinfection. One hour before induction of anesthesia, mice received a subcutaneous injection of buprenorphine at 0.1 mg/kg body weight. Induction was initiated by placing mice in an induction chamber with 5% isoflurane in oxygen (flow rate 1 L/min) until loss of righting reflex was observed. Mice were then transferred to the surgical table and switched to nose cone delivery, and the isoflurane concentration was adjusted to 1% for maintenance, with simultaneous monitoring of respiration and use of a heating blanket. A 3-mm incision was made anterior to the knee joint to expose the femoral intercondylar fossa. A 0.45-mm syringe needle was used to access the femoral bone marrow cavity, followed by expansion with a 0.7-mm needle. Using a microsyringe, 10 μL of *S. aureus* (10^6^ CFU/mL) was injected into the cavity. Control mice received 10 μL of sterile phosphate-buffered saline (PBS). Wound closure was achieved with bone wax and sutures (27). Postoperatively, prewarmed saline was administered subcutaneously at 1 mL/20 g body weight.

### 2.3 H&E staining

After a 2-week period, mice received subcutaneous buprenorphine (0.1 mg/kg body weight). After a 10-min interval, trained personnel performed euthanasia by cervical dislocation, and femurs were harvested and fixed in 4% paraformaldehyde for 48 h, followed by decalcification in 10% EDTA (pH 7.4) for 14–21 days at 4°C. Decalcified bones were dehydrated through graded ethanol, cleared in xylene, and embedded in paraffin. Serial sections (4 μm) were dewaxed in xylene and rehydrated through a descending ethanol series. Sections were stained with Mayer’s hematoxylin for 5 min, differentiated in 1% acid alcohol, rinsed in tap water, and counterstained with eosin Y for 2 min. Slides were dehydrated in ethanol, cleared in xylene, and mounted with neutral balsam for histopathological examination.

### 2.4 Micro-computed tomography analysis

Fixed femurs were dehydrated in 70% ethanol and secured in cylindrical scanning holders with longitudinal orientation. The operated femurs were scanned at an isotropic voxel size of 15 μm. Following image acquisition, bone parameters from slices 100–500 proximal to the femoral condyles were analyzed using μCT Evaluation Program V6.6 to evaluate cortical bone destruction following osteomyelitis.

### 2.5 Flow cytometry assay

Bone marrow cells were flushed with ice-cold PBS. Cells were filtered through a 70-μm cell strainer to obtain a single-cell suspension and then lysed with red blood cell lysis buffer for 5 min at room temperature. After washing with PBS containing 2% FBS, cells were counted and resuspended in staining buffer (PBS with 2% FBS and 0.1% NaN_3_) at 1 × 10^6^ cells/100 μL. Cells were incubated with Zombie NIR (1:1,000 dilution) for 15 min at room temperature in the dark. Fc receptors were blocked with anti-CD16/32 for 10 min, followed by incubation with fluorochrome-conjugated surface antibodies (anti-CD45, anti-CD11b, anti-F4/80, anti-CD63) for 30 min at 4°C. For intracellular staining, cells were fixed with Fixation/Permeabilization buffer for 20 min at room temperature, washed with permeabilization buffer, and blocked with 2% normal mouse serum. Cells were stained with antibodies (anti-SDC4, anti-ARG1, anti-MIF) for 30 min at room temperature. For intracellular cytokine staining, cells were stimulated with PMA/ionomycin and brefeldin A for 4 h, fixed/permeabilized, and stained with antibodies (anti-CXCL1, anti-CCL4). Samples were washed and resuspended in PBS for analysis. Dead cells were excluded based on viability dye fluorescence, and single cells were gated for subsequent analysis using FlowJo software.

### 2.6 Single-cell RNA sequencing and data processing

Bone marrow cells from femurs were isolated via Dulbecco’s modified Eagle’s medium (DMEM) flushing yielded single-cell suspensions for downstream scRNA-seq analysis. The Chromium Single Cell 3′ Reagent Kit of the Chromium platform (10X Genomics, Pleasanton, CA) was used for sequencing in reference to the manual. The library preparation was carried out, and the sequencing was executed on the Illumina NextSeq 500 system (Illumina, San Diego, CA, USA). Sequencing data were deposited in the GEO database with series number GSE311208.

Raw fastq data were processed with CellRanger (v7.1.0) for alignment and unique molecular identifier (UMI) counting. Cells failing quality control thresholds were eliminated according to: mitochondrial gene percentage < 20% and detected genes per cell = 500–5,000. Scrublet (v3.0.0) was used for doublet removal. Data normalization and scaling were performed with NormalizaData function of Seurat (V5.2.0). Using the 3,000 most variable genes, we performed principal component analysis (PCA) and visualized the data with uniform manifold approximation and projection (UMAP) based on the top 15 components. Using FindAllMarkers (log fold change threshold = 1; min. pct = 0.25), cluster-specific markers were identified. These clusters were then manually annotated by consulting the CellMarker 2.0 database (28). The cell proportion of each sample was calculated and plotted with the “cellRatioPlot” function of the scRNAtoolVis (v0.1.0) package.

### 2.7 Construction of single-cell trajectories

Monocle3 (v1.0.0) was utilized to perform single-cell trajectory analysis. For the trajectory analysis, cells were ordered in pseudo-time based on genes that satisfied the criteria of mean_expression ≥ 0.08 and dispersion_empirical ≥ 1 * dispersion_fit, as determined by Monocle3. Dimensionality reduction was achieved using the reduceDimension function with the settings reduction_method = “DDRTree” and max_components = 3. The pseudo-time of each cell was calculated with the “orderCells” function. Minimum spanning trees were visualized with plot_cell_trajectory. Pseudo-time dynamic genes (*q*-val < 10^−5^), identified by differentialGeneTest, were displayed on plot_pseudotime_heatmap and grouped by expression pattern.

### 2.8 Transcription factors and regulon activity analysis

PySCENIC was designed to evaluate the activity of transcription factors (TFs) within individual cells (29). By examining the co-expression of genes related to a specific transcription factor, the activity of that transcription factor could be inferred. In PySCENIC, the GRNBoost algorithm was used to calculate the weights between transcription factors and their target genes, with count data as the input. Then, the output of PySCENIC was imported into R, and AUCell and SCENIC were used to calculate the average transcription factor activity.

### 2.9 Cell–cell interaction analysis with CellChat

The CellChat (v1.6.1) package was utilized to explore the communication probabilities between the macrophages of each subtype and all the other immune cells. The mouse CellChatDB was used, and we focused on the secreted signaling pathways. The expression of ligand–receptor pairs of the CellChatDB was calculated for the cell interaction quantification, and the interaction networks were constructed (16).

### 2.10 Statistical analysis

We conducted all statistical analyses using the Python (v3.13.0) and R (v4.4.2) platforms. The implementation of tests included two-sided unpaired Student’s *t*-tests and unpaired Wilcoxon rank-sum tests where appropriate. A *p*-value less than 0.05 was considered statistically significant.

## 3 Results

### 3.1 Dynamic gene expression landscape of osteomyelitis

**Figure 1:**
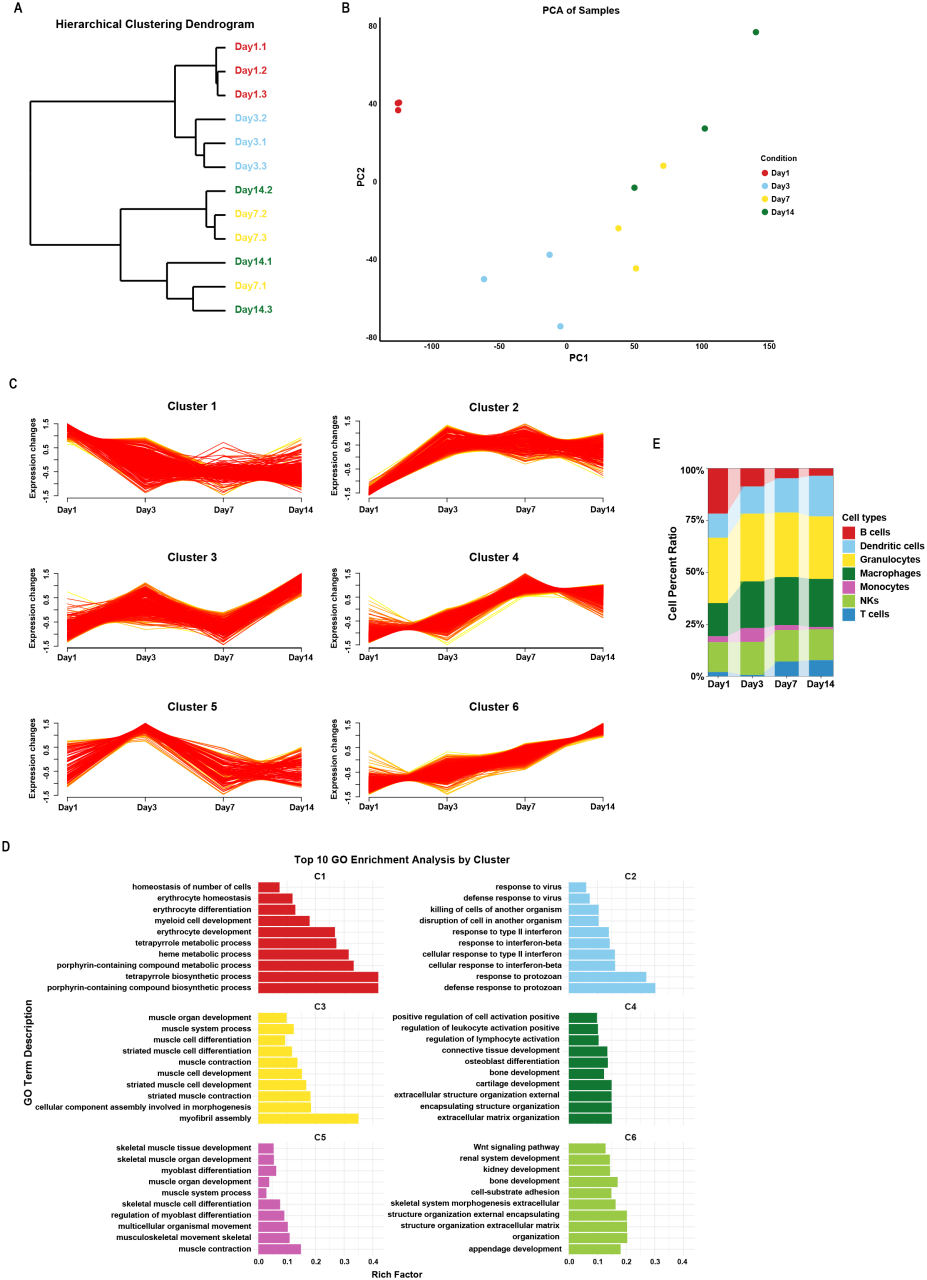
Time-series RNA-seq analysis of mouse osteomyelitis samples. Hierarchical clustering **(A)** and PCA analysis **(B)** showing the correlation of samples of different time points. Genes with similar expression patterns across time were clustered into six gene clusters **(C)** and their biological functions were explored through GO biological process enrichment analysis **(D)**. **(E)** Immune infiltration analysis revealed the proportions of major immune cell populations at different time points.

Hierarchical clustering of GSE168896 revealed that day 1/day 3 samples clustered together, while day 7/day 14 samples formed a separate branch, indicating distinct osteomyelitis stages (**Figure 1A**). PCA confirmed this clustering pattern (**Figure 1B**). Based on these results, day 1/day 3 was classified as the acute phase, while day 7/day 14 was classified as the subacute phase (27, 30). A total of 2,382 DEGs across time points (day 3 vs. day 1, day 7 vs. day 3, day 14 vs. day 7) were used for soft clustering analysis, identifying six gene clusters. Notably, cluster 1 (C1) showed a decreasing trend from day 3 and had a low expression level, functionally associated with erythrocyte development. C2 was upregulated on day 3 after osteomyelitis modeling and maintained at a high expression level thereafter, and its function was related to the response to interferon. C3 expression fluctuated in the progression of osteomyelitis. C4, containing the *Il6*, C-X-C motif chemokine ligand 5 (*Cxcl5*), and *Cxcl10*, showed increased expression from day 7 onward and was associated with leukocyte activation and ossification. C5 showed a high expression level on day 1/day 3 and decreased in day 7/day 14, functionally associated with myoblast differentiation. C6 containing *Il17a*, *Il23r*, RANKL (*Tnfsf11*), osteoclast-associated transmembrane protein (Osterm, or Ostap), Osteoclast stimulatory transmembrane protein (Ocstamp), and TNF receptor superfamily member 19 (*Tnfrsf19*) exhibited a progressive increase in expression, indicating its role in bone development and the Wnt signaling pathway (**Figures 1C, D**). DEGs of each cluster and GO enrichment are shown in **Supplementary Table 1**. Immune cell infiltration analysis revealed significant increases in macrophages, dendritic cells (DCs), and NK cells on day 3, while B cells showed a progressive decline (**Figure 1E**).

### 3.2 Single-cell mapping of osteomyelitis-driven immune reprogramming in bone marrow

**Figure 2:**
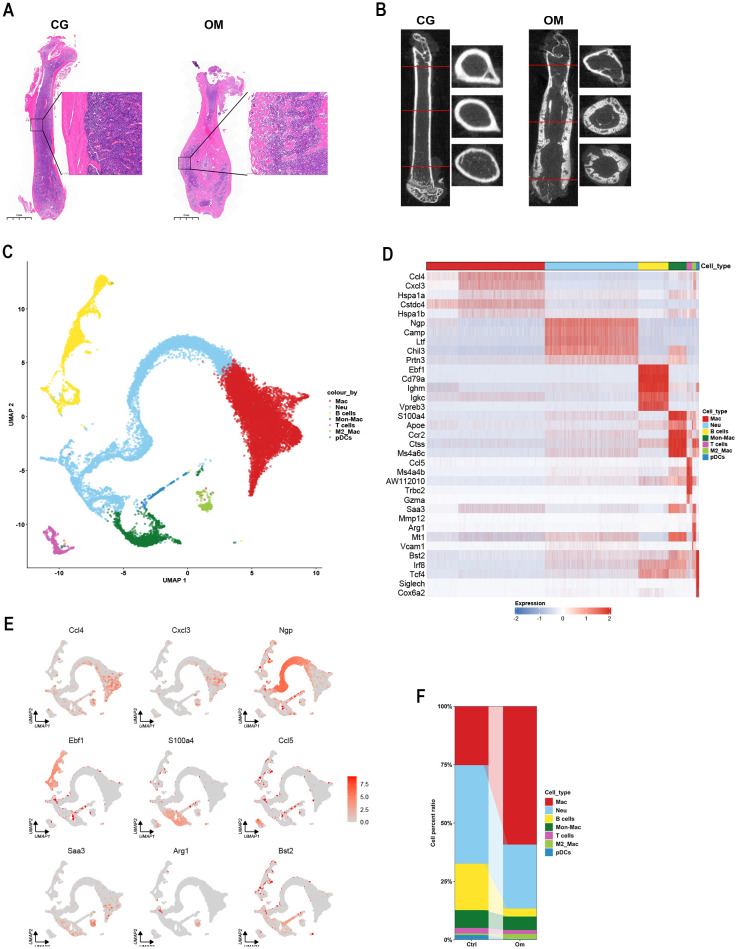
Single-cell RNA-seq analysis. Identification of the osteomyelitis model by H&E staining **(A)** and micro-CT **(B)**. H&E staining revealed diffuse infiltration of numerous neutrophils within the marrow cavity of the femur in osteomyelitis mice, with localized abscess formation, accompanied by necrosis of bone trabeculae and marrow tissue, and micro-CT revealed focal osteolysis, cortical bone discontinuity, and sequestrum formation in the femurs of mice with osteomyelitis. **(C)** UMAP showed the immune cell clusters in the control and the osteomyelitis samples. **(D)** The heatmap showed cell markers of each cell cluster. **(E)** The expression of the most highly expressed genes of each cell cluster. **(F)** Cell percent ratio of the ctrl and the osteomyelitis samples.

To investigate the in-depth immune mechanisms of osteomyelitis, we established a mouse model of osteomyelitis, and the successful model construction was confirmed by H&E staining (**Figure 2A**) and micro-computed tomography (micro-CT) (**Figure 2B**). Single-cell sequencing analysis was used to explore new knowledge. Results showed that the bone marrow cell distribution of mice with osteomyelitis is consistent with that of control mice (**Supplementary Figure 1A**). Furthermore, single-cell sequencing analysis identified 12 cellular subpopulations (C0–C11) (**Supplementary Figure 1B**). The marker genes of C0 contained interferon-stimulated gene 15 (*Isg15*), *Cxcl3*, *Ccl4*, and other inflammatory genes in macrophages. Marker genes in C2 include colony-stimulating factor 1 (*Csf1*) and cluster of differentiation 63 (*Cd63*), which were marker molecules of macrophages. Therefore, C0 and C2 were identified as macrophage subpopulations. C1 contained neutrophil granule protein (*Ngp*) and lactotransferrin (*Ltf*), which were classical markers of neutrophils, and C4 contained the Ltf and neutrophil-specific gene cathelicidin antimicrobial peptide (*Camp*), C5 contained elastase (*Elane*) and proteinase 3 (*Prtn3*), and C7 contained stathmin 1 (*Stmn1*) and *Prtn3*, which were associated with neutrophil maturation and antimicrobial activity, so C1, C4, C5, and C7 were identified as neutrophils. In C3, *Cd79a* and *Cd79b* were classical markers of B cells, and membrane spanning 4-domains A1 (*Ms4a1*) was a marker of mature B cells. In C8, V-set pre-B-cell surrogate light chain 1 (*Vpreb1*), *Vpreb3*, and immunoglobulin lambda-like polypeptide 1 (*Igll1*) were markers of early B cells (pre-B cells). So, C3 and C8 were identified as a B-cell subgroup. C6 contained monocyte-/macrophage-associated genes, such as S100 calcium-binding protein A4 (*S100a4*), apolipoprotein E (*Apoe*), and cathepsin S (*Ctss*). In C9, *Cd3g* and T-cell receptor beta constant 2 (*Trbc2*) were components of the T-cell receptor complex, and *Cd8a* suggests that C9 was identified as cytotoxic T cells. In C10, arginase 1 (*Arg1*), macrophage scavenger receptor 1 (*Msr1*), and *Cd38* were markers of M2 macrophages, and C10 was then identified as M2-like macrophages. C11 contained the marker genes Siglech and interferon regulatory factor 8 (*Irf8*), which are specific markers of plasmacytoid DCs (pDCs), so C11 was identified as pDCs (**Figure 2C**). Marker genes per cell cluster were visualized in heatmaps (**Figures 2D, E**; **Supplementary Table 2**). The proportion of macrophage infiltration increased most significantly after osteomyelitis and showed the highest proportion among immune cells in osteomyelitis (**Figure 2F**). This is similar to the results of the dataset GSE168896 (**Figure 1E**).

### 3.3 Characteristics of monocytes/macrophages

**Figure 3:**
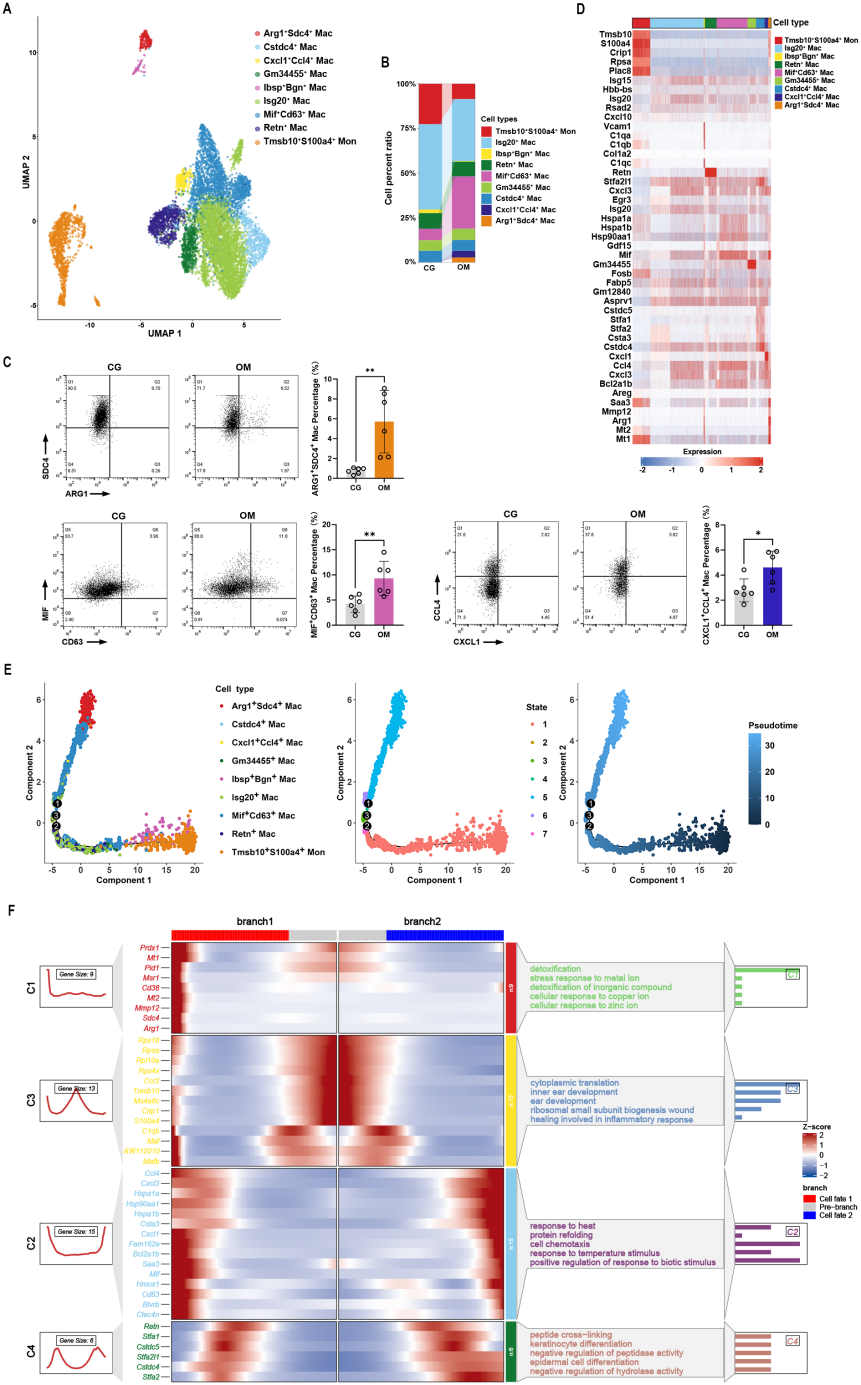
Monocyte/macrophage subcluster analysis. Cell clusters **(A)** and cell percent ratio **(B)** of the control and the osteomyelitis samples. **(C)** Macrophage subtypes were identified by flow cytometry. **(D)** Cell markers of each sample. **(E)** Pseudo-time analysis of all the monocyte/macrophage cell clusters. **(F)** Heatmap and function annotation of differentially expressed genes at branch point 1. *: *p* < 0.05; **: *p* < 0.01.

As M2 macrophages showed a core regulatory role in osteomyelitis progression, we then explored the functions of monocyte/macrophage subtypes. The distribution of monocytes/macrophages in osteomyelitis mice was consistent with that in the control mice (**Supplementary Figure 2**). Nine subclusters of monocytes/macrophages were identified and renamed with their marker genes (**Figure 3A**; **Supplementary Table 3**). M2 macrophages had two distinct subpopulations, one highly expressing *Arg1* and Syndecan-4 (*Sdc4*) and the other highly expressing integrin binding sialoprotein (*Ibsp*) and Biglycan (*Bgn*). Thus, M2 macrophages were subdivided into two subpopulations: Arg1^+^Sdc4^+^ macrophages and Ibsp^+^Bgn^+^ macrophages (**Figure 3A**). The infiltration of Mif^+^Cd63^+^, Cxcl1^+^Ccl4^+^, and Arg1^+^Sdc4^+^ macrophages increased dramatically after osteomyelitis (**Figure 3B**). These results were also validated by flow cytometry (**Figure 3C**). Markers of each subtype were shown in a heatmap (**Figure 3D**). Pseudo-time analysis suggested that Tmsb10^+^S100a4^+^ monocytes were at the start point of development, and Arg1^+^Sdc4^+^ and Mif^+^Cd63^+^ macrophage subtypes were fully differentiated (**Figure 3E**). At branch point 1, monocyte/macrophage cells were differentiated to Arg1^+^Sdc4^+^ and Mif^+^Cd63^+^ macrophages in branch 1, and the differentiated marker genes were expressed, such as *Msr1*, *Cd38*, *Mmp12*, and *Arg1* (**Figure 3F**).

### 3.4 Identification of potential regulons of each cell subtype

**Figure 4:**
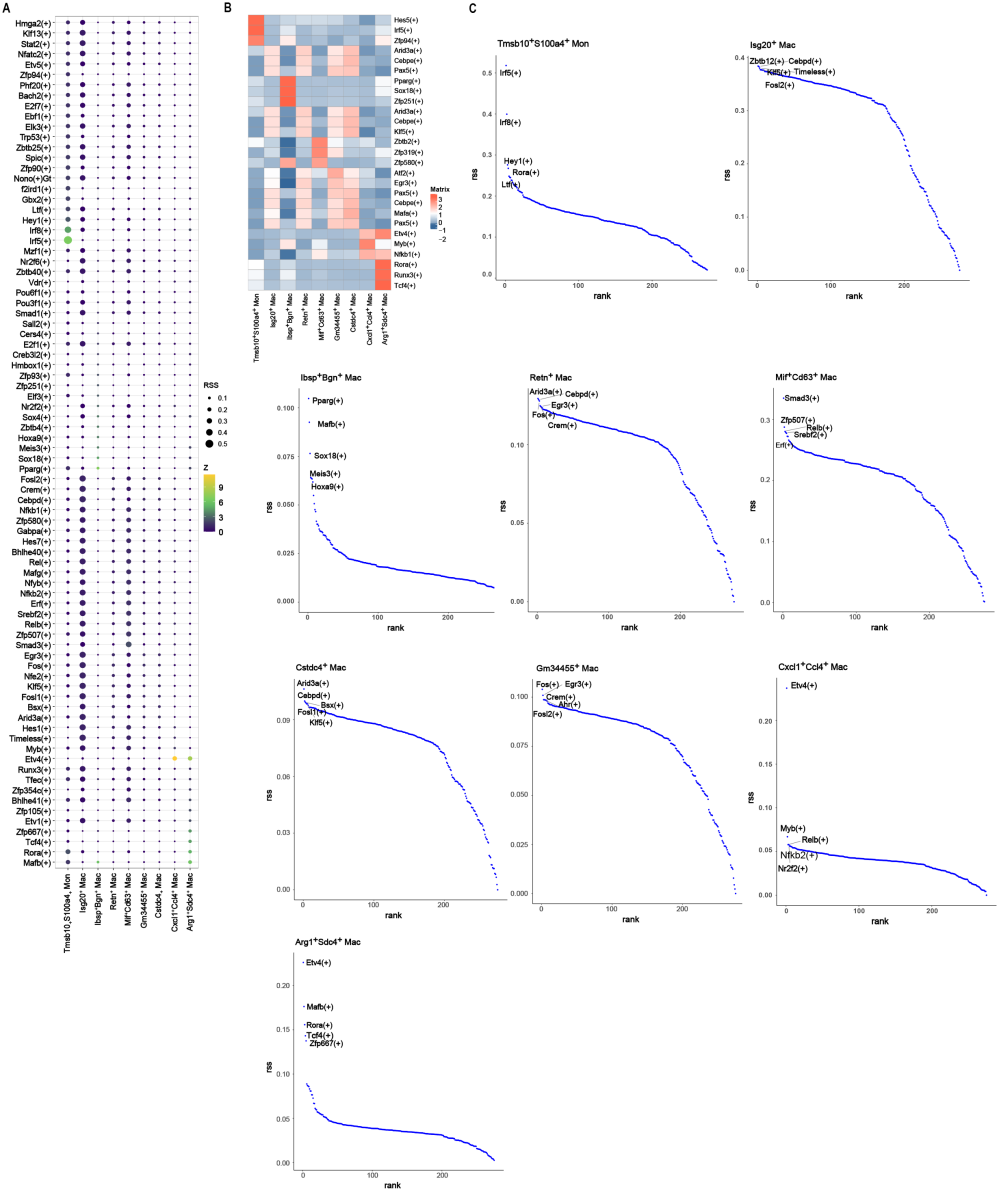
Transcription factor analysis. All the activated transcription factors in immune cell clusters **(A)**, the differentially activated transcription factors **(B)**, and the most activated transcription factors of each cell cluster **(C)**.

SCENIC-based regulon profiling pinpoints the core transcriptional switches that lock each cell type into its identity and assembles the corresponding gene-regulatory circuitry. The output revealed distinct, lineage-specific regulons proposed to safeguard cellular phenotypes (**Figure 4A**). We then converted regulon activity into binary on/off calls and mapped these signatures onto individual cell clusters (**Figure 4B**). ETS variant transcription factor 4 (Etv4) and RAR-related orphan receptor A (Rora) emerged as the most specific regulators within Arg1^+^Sdc4^+^ macrophages. In Cxcl1^+^Ccl4^+^ macrophages, these were Etv4 and V-Myb avian myeloblastosis viral oncogene homolog (Myb) (**Figure 4C**). These data revealed distinct regulon landscapes across the cell types.

### 3.5 Macrophage subtypes interact with other immune cells through ligand–receptor interactions

**Figure 5:**
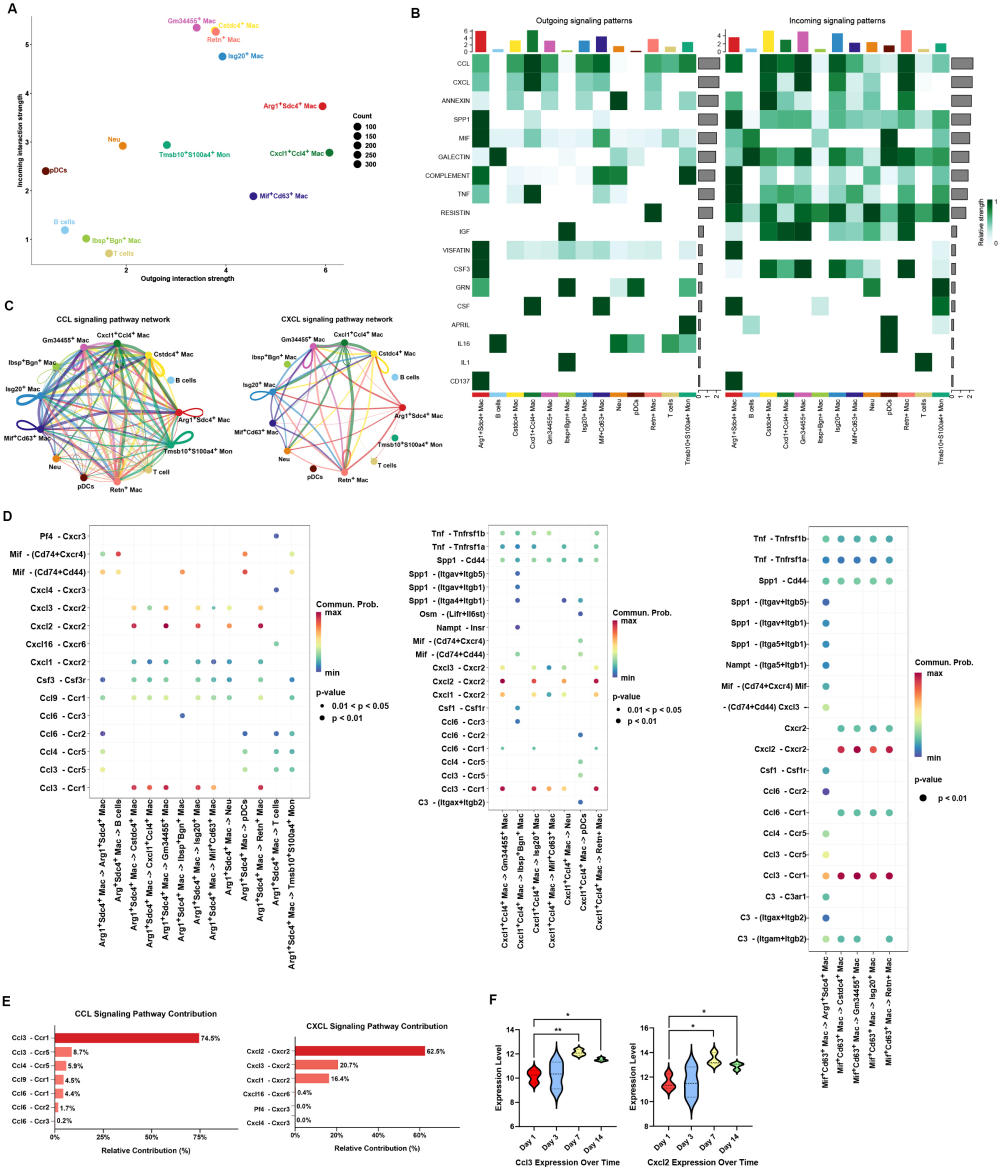
CellChat analysis. **(A)** Incoming and outgoing strength of cell clusters. **(B)** The incoming and outgoing signaling patterns of cell clusters. **(C)** CCL and CXCL signaling pathway network; secreted signaling interactions of cell clusters **(D)**. **(E)** Relative contribution of each L–R pair in the CCL and CXCL signaling pathway. **(F)** Expression of Ccl3 and Cxcl2 at different time points. *: *p* < 0.05; **: *p* < 0.01.

For CellChat analysis, the Arg1^+^Sdc4^+^, Cxcl1^+^Ccl4^+^, and Mif^+^Cd63^+^ macrophages showed the most outgoing interactions with other immune cells (**Figures 5A, B**). The CCL and CXCL signaling pathways were among the most active mediators of intercellular communication (**Figures 5B, C**). Cxcl2–Cxcr2 and Ccl3–Ccr1 ligand–receptors contributed mostly in Arg1^+^Sdc4^+^, Cxcl1^+^Ccl4^+^, and Mif^+^Cd63^+^ macrophages (**Figures 5D, E**), indicating the key role of Ccl3–Ccr1 and Cxcl2–Cxcr2 in them. In the dataset GSE168896, Ccl3 and Cxcl2 appeared in cluster 4 (**Supplementary Table 1**) and increased with the extension of time (**Figure 5F**). This further suggested that Ccl3–Ccr1 and Cxcl2–Cxcr2 may play key roles in osteomyelitis through macrophages.

## 4 Discussion and conclusion

Osteomyelitis often leads to severe bone destruction and inflammation of surrounding tissues, resulting in prolonged treatment cycles and limb dysfunction. Post-infection immune remodeling stalls repair and fuels bone loss. Next-generation sequencing unveiled the immune landscape reshaped by osteomyelitis. By integrating bulk and single-cell RNA-seq, we pinpointed how host defenses both shape and are shaped by this infection, while revealing tractable drug targets.

Neutrophils, macrophages, and B and T cells are rapidly recruited to the site of osteomyelitis. Their relative abundance and activation states undergo dynamic changes as the disease progresses and the local microenvironment evolves (31). Monocytes and macrophages, for instance, serve as frontline innate sentinels that engulf and eliminate invading microbes (32). T cells coordinate adaptive immune responses, while B cells produce antibodies targeting specific pathogens—a dual mechanism essential for effective host defense. In osteomyelitis, the presence of lymphocytes indicates a prolonged immune reaction (33). Immune cells secrete cytokines and chemokines, which are signaling molecules that mediate inflammation and recruit other immune cells to the infected site. Osteomyelitis is marked by a surge of pro-inflammatory mediators (34). Analysis of immune cell infiltration in this study revealed an increase in DCs and macrophages following osteomyelitis. Notably, monocytes/macrophages exhibited the most significant expansion among these cell populations, underscoring their central role in the disease.

Following injury, monocytes and macrophages coordinate tissue repair by modulating inflammation, clearing cellular debris, and secreting growth factors that promote healing (35). We delineated nine transcriptionally distinct subsets of these cells and characterized how osteomyelitis dynamically reshapes each one’s molecular profile. One of the major subsets was the M2-like Arg1^+^Sdc4^+^ macrophages, highly expressing key genes including *Arg1* and *Mmp12*. Arg1^+^ macrophages, characterized by the expression of Arg1, metabolize L-arginine into urea and L-ornithine (36), inhibiting the production of nitric oxide (NO) and alleviating inflammatory responses (37). L-ornithine feeds polyamine synthesis, a key step for tissue repair and regeneration (38). By producing L-ornithine, Arg1^+^ macrophages promote the synthesis of polyamines, contributing to tissue repair and regeneration (39). These macrophages also secrete cytokines and growth factors, which further facilitate tissue repair (40). Furthermore, Arg1^+^ macrophages regulated the metabolism of L-arginine, thereby influencing the functions of other immune cells. For instance, they can limit the activation of T cells, thereby suppressing excessive immune responses (41). Although Arg1^+^ macrophages are primarily associated with anti-inflammatory and tissue repair functions, they also participate in the clearance of pathogens and cellular debris under certain circumstances. For example, in some chronic infections, these macrophages can clear dead cells and pathogens through phagocytosis (35). Sdc4^+^ macrophages participated in inflammation by shaping the release of immune mediators. In atherosclerosis models, the reduction of Sdc4 exacerbates the pro-inflammatory capacity of macrophages (42). In our study, Arg1^+^Sdc4^+^ macrophages increased after osteomyelitis, suggesting that these macrophages tip the balance between resolution and persistence of osteomyelitis.

Cxcl1^+^Ccl4^+^ macrophages were another increased subpopulation of macrophages that emerged in osteomyelitis. CXCL1 is a significant CXCL chemokine that attracts neutrophils and other immune cells to the sites of inflammation. Cxcl1^+^ macrophages amplified inflammation by releasing CXCL1 to recruit neutrophils to the tumor microenvironment. Additionally, Cxcl1^+^ macrophages secreted other inflammatory mediators, which further modulated the inflammatory response (43). Interestingly, Cxcl1^+^ macrophages recruited myeloid-derived suppressor cells (MDSCs) and regulatory T cells (Tregs), weaving a local immunosuppressive network that shields tumor cells from immune attack (44, 45). These results all confirm the multiple roles of Cxcl1^+^ macrophages in the inflammatory response. CCL4 could recruit monocytes, T cells, and other immune cells to inflamed sites. Ccl4^+^ macrophages enhanced the inflammatory response by secreting Ccl4, which promoted the recruitment of these immune cells (46). Ccl4^+^ macrophages also secreted other inflammatory mediators (47). Similarly, CCL4 attracted MDSCs and Tregs, contributing to the creation of a local immunosuppressive milieu (46). Thus, in osteomyelitis, Cxcl1^+^Ccl4^+^ macrophages seed the inflamed bone with CXCL1 and CCL4, recruiting immune cells that both push the reaction forward and keep it from fading.

Mif^+^Cd63^+^ macrophages significantly increased in the osteomyelitis mouse model in this study. MIF is an important inflammatory mediator that can promote the recruitment and activation of inflammatory cells. Meanwhile, tumor cells reshape tumor-associated macrophages (TAMs) into an immunosuppressive APOE^+^ subset through the MIF–CD74 axis, thereby driving tumor progression (48). In hepatocellular carcinoma (HCC), CD63^+^ TAMs promoted the proliferation, invasion, and metastasis of HCC cells by inducing epithelial–mesenchymal transition (EMT) and lipid reprogramming (49). As a signature gene of the inflammatory expression program, CD63 was associated with poor prognosis in patients with HCC (49). Exosomal CD63 is involved in the intracellular transport of pathogens to host cells (50). In osteomyelitis, Mif^+^Cd63^+^ macrophages might be responsible for pro-inflammation, and more research is needed to confirm this.

The Ccl3 (also known as MIP-1α) and its primary receptor Ccr1 signaling axis constitute a pivotal pathway regulating immune cell migration and activation. This pathway plays a critical role in diverse pathophysiological processes, including inflammation, infection, autoimmune diseases, and tumor microenvironment modulation (51). Ccl3 is produced by a variety of cells, including activated macrophages and lymphocytes. By binding to Ccr1, it efficiently recruits monocytes/macrophages, neutrophils, DCs, and T cells to sites of inflammation or injury (52). Ccl3 frequently interacts synergistically or antagonistically with pathways such as Cxcl2–Cxcr2, thereby forming a complex regulatory network of chemokines (52). CCL3 exerts its function by promoting the recruitment of leukocytes to inflammatory sites and is closely related to immune surveillance and tolerance. Currently, CCL3 has become a prognostic biomarker for both solid tumors and hematological malignancies (53). Therefore, in osteomyelitis, the Ccl3–Ccr1 axis may lead to extensive inflammatory cell infiltration within the bone marrow cavity and contribute to persistent infection, thereby facilitating its progression into chronic osteomyelitis.

The Cxcl2–Cxcr2 pathway mediates communication between immune cells, notably macrophages, indicating potential regulation of osteomyelitis. CXCL2 is expressed in macrophages, endothelial cells, and tumor cells and recruits and activates neutrophils through the Cxcl2–Cxcr2 axis, which is essential for host defense against infection and tissue repair (54, 55). Cxcl2 recruited MDSCs and neutrophils through Cxcr2, forming an immunosuppressive microenvironment that promoted tumor progression. In colorectal cancer, CXCL2 levels rose alongside M2-polarized macrophage numbers, a liaison that fostered tumor spread and immune escape (56, 57). In chronic inflammatory diseases, CXCL2 exacerbated chronic inflammation by recruiting and activating neutrophils (58, 59). Similar to the three macrophage subtypes mentioned above, the Cxcl2–Cxcr2 signaling pathway is committed to sustaining inflammation and may contribute to the high incidence of chronic osteomyelitis.

In conclusion, our study found that Arg1^+^Sdc4^+^ and Cxcl1^+^Ccl4^+^ macrophages emerged after infection, while Mif^+^Cd63^+^ macrophages showed a dramatic increase. Beyond its broad reach across immune cell types, the Cxcl2–Cxcr2 axis is especially pivotal within macrophage subsets, implicating it as a key driver of osteomyelitis progression. Our results provided a comprehensive view of the genetic and cellular dynamics in osteomyelitis, highlighting the intricate immune landscape and the potential for targeted therapies. Further research is warranted to explore these findings in clinical settings and to develop novel treatment strategies that can improve outcomes for patients with osteomyelitis.

## Data Availability

The datasets presented in this study can be found in online repositories. The names of the repository/repositories and accession number(s) can be found below: GSE311208 (GEO).

## Supplementary material

Supplementary Figure 1 UMAP showed the distribution of samples **(A)** and different immune cell clusters **(B)**.

Supplementary Figure 2 UMAP showed the distribution of samples.
