# Supplementary figures and images for: Combination of single-cell and bulk RNA-seq reveals changes in the immune landscape in osteomyelitis
Source: Front Immunol. 2026 Feb 27;17:1746323. doi: 10.3389/fimmu.2026.1746323 (PMC12982112; doi:10.3389/fimmu.2026.1746323)

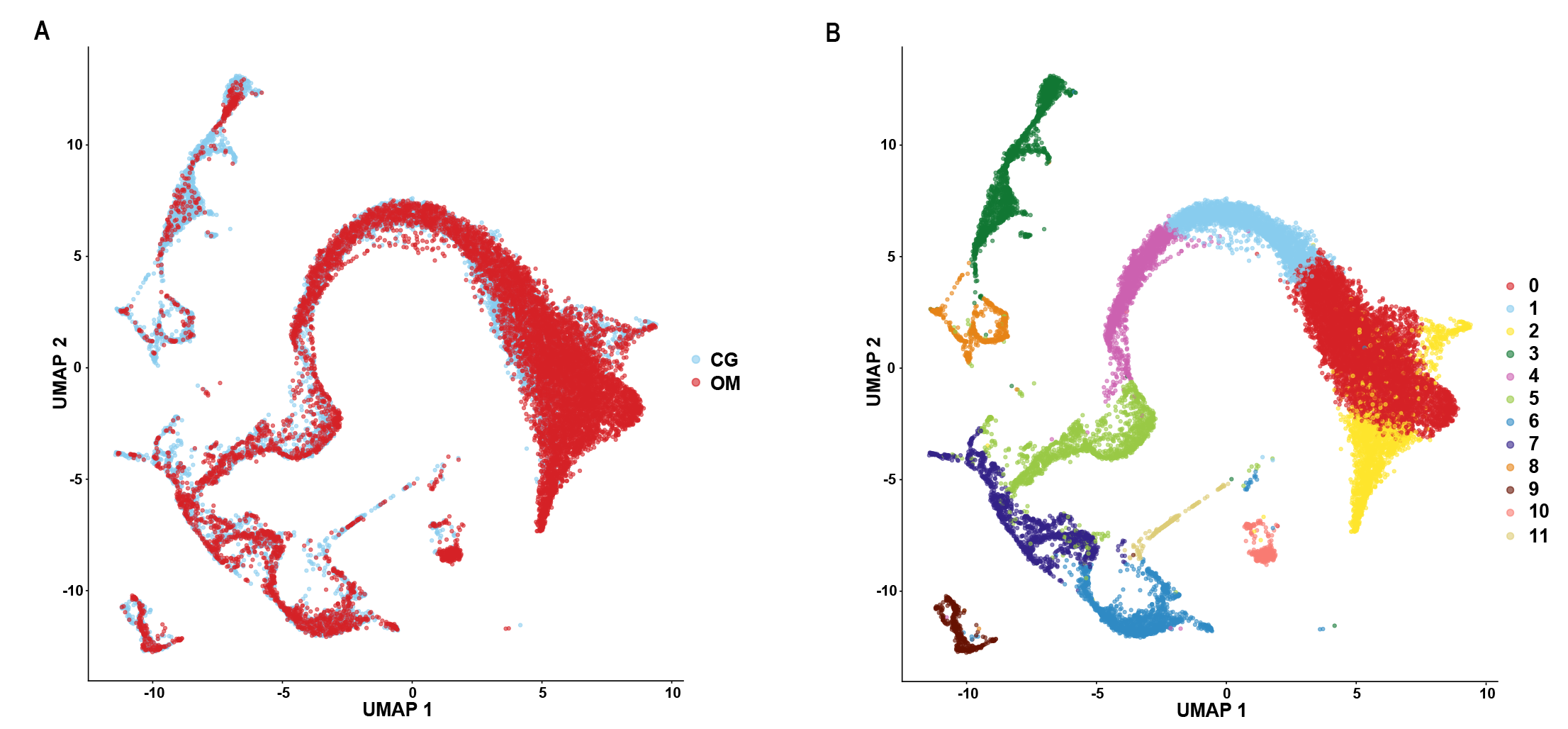

Supplement: Supplementary Figure 1 — UMAP showed the distribution of samples (A) and different immune cell clusters (B). [file Image1.tif]

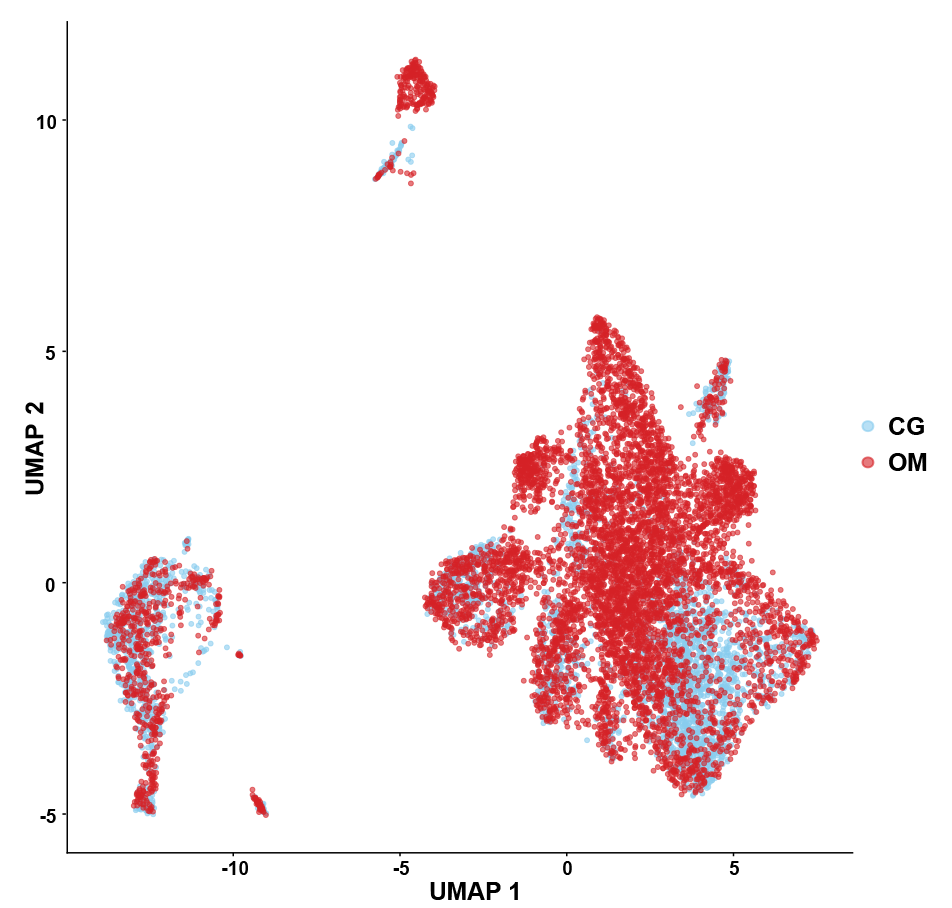

Supplement: Supplementary Figure 2 — UMAP showed the distribution of samples. [file Image2.tif]
